# consICA: an R package for robust reference-free deconvolution of multi-omics data

**DOI:** 10.1093/bioadv/vbae102

**Published:** 2024-07-13

**Authors:** Maryna Chepeleva, Tony Kaoma, Andrei Zinovyev, Reka Toth, Petr V Nazarov

**Affiliations:** Multiomics Data Science Research Group, Department of Cancer Research, Luxembourg Institute of Health, Strassen L-1445, Luxembourg; Faculty of Science, Technology and Medicine, University of Luxembourg, Esch-sur-Alzette L-4365, Luxembourg; Bioinformatics and AI Unit, Department of Medical Informatics, Luxembourg Institute of Health, Strassen L-1445, Luxembourg; In Silico R&D, Evotec, Toulouse 31100, France; Multiomics Data Science Research Group, Department of Cancer Research, Luxembourg Institute of Health, Strassen L-1445, Luxembourg; Bioinformatics and AI Unit, Department of Medical Informatics, Luxembourg Institute of Health, Strassen L-1445, Luxembourg; Multiomics Data Science Research Group, Department of Cancer Research, Luxembourg Institute of Health, Strassen L-1445, Luxembourg; Bioinformatics and AI Unit, Department of Medical Informatics, Luxembourg Institute of Health, Strassen L-1445, Luxembourg

## Abstract

**Motivation:**

Deciphering molecular signals from omics data helps understanding cellular processes and disease progression. Effective algorithms for extracting these signals are essential, with a strong emphasis on robustness and reproducibility.

**Results:**

R/Bioconductor package *consICA* implements consensus independent component analysis (ICA)—a data-driven deconvolution method to decompose heterogeneous omics data and extract features suitable for patient stratification and multimodal data integration. The method separates biologically relevant molecular signals from technical effects and provides information about the cellular composition and biological processes. Build-in annotation, survival analysis, and report generation provide useful tools for the interpretation of extracted signals. The implementation of parallel computing in the package ensures efficient analysis using modern multicore systems. The package offers a reproducible and efficient data-driven solution for the analysis of complex molecular profiles, with significant implications for cancer research.

**Availability and implementation:**

The package is implemented in R and available under MIT license at Bioconductor (https://bioconductor.org/packages/consICA) or at GitHub (https://github.com/biomod-lih/consICA).

## 1 Introduction

In cancer research, the interpretation of patient-derived molecular profiles, including gene expression and DNA methylation, can be hampered by tumour and tissue heterogeneity that masks the important molecular signatures. The complexity can arise from variations in the proportions of different cell types or from clonal divergences among tumour cell populations. Even in a single cell, multiple transcriptional programs are running at the same time. Additionally, technical biases of experimental platforms may constrain comparability between batches or datasets. Matrix factorization approaches are widely applied to ‘omics’ data to decipher hidden signals in biological data. Independent component analysis (ICA) is a powerful unsupervised matrix factorization method used for deconvolution of mixed signals. ICA captures signals as mutually statistically independent as possible (Hyvarinen and Oja 2000, Sompairac *et al.* 2019). However, ICA's sensitivity to the initial estimates may lead to certain stochasticity of the results that reduces its reproducibility and may lead to misinterpretations when comparing different datasets. To address this issue, we previously proposed a consensus approach that performs multiple runs and combines results into a robust decomposition (Nazarov *et al.* 2019).

Here, we present *consICA*, an R Bioconductor package that implements consensus ICA as a robust deconvolution approach, making it fully compatible with the Bioconductor environment. Supplementary functionality augments the core method, facilitating a comprehensive analysis and enhancing the overall utility. The package benefits from parallel calculation and can analyse both single-cell and bulk-sample molecular data to extract meaningful biological signals. The features and limitations of other tools for ICA deconvolution are described in Supplementary Table S1, and algorithm comparison, including running time, is given in Supplementary Text S1. Existing R packages for ICA deconvolution, such as *MineICA* or *IPCA*, just provide the interface to *fastICA—*a standard implementation of the ICA, which lacks reproducibility. Stable approaches, such as *robustica* and *StabilizedICA* (used in the BIODICA framework) are implemented only in Python and cannot be easily integrated into R code. Contrary to these approaches, we used an alternative way to build stable components: mapping and averaging components instead of clustering multiple results. Additionally, *consICA* provides extended functionality that includes automatic functional annotation of the extracted signals, linking component weights to experimental factors by ANOVA, survival analysis by Cox regression, variance analysis, and automatic reporting for a fast understanding of the results. The method was successfully applied in the published studies to bulk-sample RNA-seq (Dirkse *et al.* 2019, Golebiewska *et al.* 2020), single-cell RNA-seq (Dirkse *et al.* 2019) and methylation data (Scherer *et al.* 2020).

## 2 Methods

**Figure 1.**
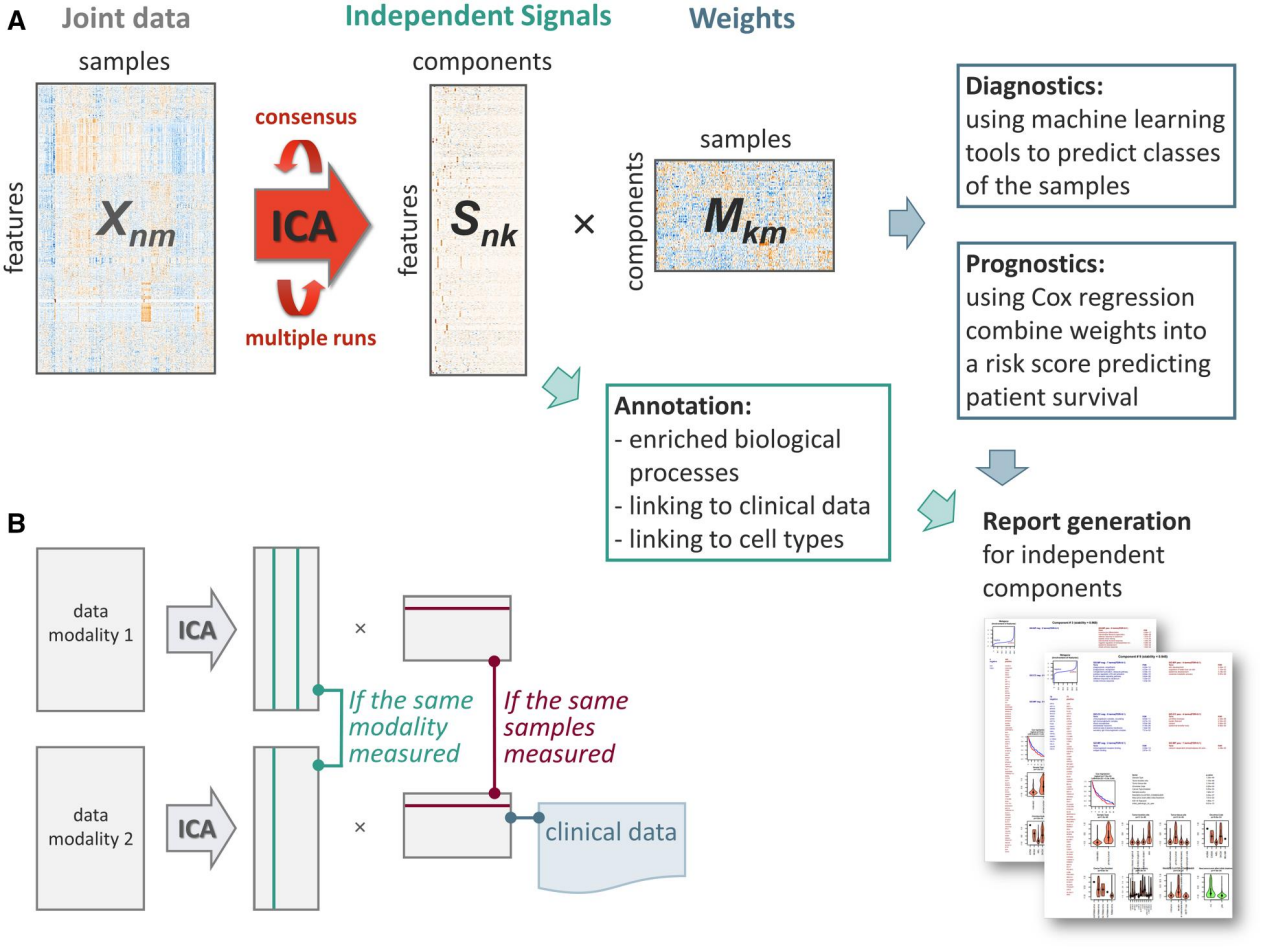
The *consICA* approach: a schematic representation. *consICA* allows the deconvolution of various modalities: bulk or single-cell; transcriptomics, epigenomics, miRNAs, proteomics, etc.; combined data of reference (e.g. TCGA) and investigation (e.g. new patients) data sets. (A) Independent component analysis (ICA) decomposes the combined matrix into independent signals (*S*) and their weights (*M*). Independent components could be annotated with biological processes/cell subtypes, while *M* could be linked to patient groups and patient survival. Automatic report accumulates all information of independent components. (B) For data integration, the correlation analysis of independent components or their weights can be used.

The results of a single ICA run depend on the initial estimations. Robust decomposition is reached here by averaging the results of multiple ICA runs with different initial estimations. At each run, the scaled initial data matrix ***X_nm _***gene expression, DNA methylation, or other omics datasets with *n* features measured over *m* samples, is presented as a matrix product of independent signals ***S_nk_*** and their weights ***M_km_*** (Fig. 1A). We optimized computations and efficiently implemented basic ICA (Hyvarinen and Oja 2000, Marchini *et al.* 2023) for multiple parallelized runs. The obtained components have random order and sign, and should therefore be matched by the correlation of ***S*** columns, reordered, redirected, and averaged into consensus matrices ***S*** and ***M*** (Captier *et al.* 2022).

The resulting matrix ***S*** contains only a few strongly contributing values (positive or negative) in each column, while the majority of values are around zero. In gene expression analysis, the columns of ***S*** can be functionally annotated using over-representation or gene-set enrichment analysis (Subramanian *et al.* 2005, Alexa and Rahnenfuhrer 2023). Importantly, the positively and negatively contributing genes are annotated separately, as in most cases, only one direction of an independent component has a pronounced biological meaning. Weight matrix ***M*** is linked to clinical data using ANOVA and Cox regression. The weights can also be considered as engineered features for machine learning algorithms. Importantly, some components isolate experimental batch effects or platform biases, removing their effects from biological signals. Such components can either be excluded from further analysis or removed by setting their weights to zero and reconstructing the scaled data matrix.

*consICA* can be applied to several omics data, obtained on the same samples. Then, data integration can be performed by linking components across omics levels, or by correlating the rows of corresponding ***M*** matrices (Fig. 1B). The same approach can be used to connect deconvolved omics signals to unstructured data in multi-modal analysis (e.g. correlating rows of ***M*** with embeddings, obtained from histopathological images by a deep learning model).

The functions included in the *consICA* package enable the interpretation of components: functional annotation of components with gene ontology (GO) terms, association of component weights to experimental variables and survival using ANOVA and Cox regression accordingly. In addition, *consICA* provides automatic report generation, describing each component in terms of GO and highlighting its linkage to factors or patient survival. These reports simplify analysis for users and are helpful for quick investigating of the components’ content, aiding to choose an optimal number of components. Another important aspect to consider when estimating the number of components is dataset size; we recommend not exceeding one-third of the total number of samples.

## 3 Results

The developed *consICA* method was initially validated on *in vitro* mixed samples of SEQC (Consortium 2014), showing good separation of *in vitro* mixed transcriptomes (Supplementary Fig. S1). Below we provide examples of successful applications of our algorithm and *consICA* package for specific tasks, including (i) correction of technical biases or covariates, (ii) extraction of biological signals and proportions of cell subtypes that could be used as predictive features, and (iii) multi-omics and multi-modal data integration.

### 3.1 *consICA* to correct technical biases

The batch effect between in-house mRNA/miRNA data of melanoma patients and TCGA-SKCM data were successfully corrected by *consICA* in Nazarov *et al.* (2019). Similarly, we mapped transcriptomes of in-house glioblastoma cell lines and patient tissues to the TCGA-GBM data in Golebiewska *et al.* (2020). Supplementary Fig. S2A and B. *consICA* also helped to correct a batch effect in an unbalanced single-cell RNA-seq dataset by isolating it in one of the independent components and removing it as described in Section 2. The batch-corrected expression matrix was used for downstream analysis (Dirkse *et al.* 2019). Finally, we used *consICA* as a part of *DecompPipeline*, a tool aimed at precise reference-free deconvolution of DNA methylation data. There, we removed sex, age and ethnicity effects from the data, improving the identification of tumour- and stroma-related signals (Scherer *et al.* 2020).

### 3.2 *consICA* to identify biologically relevant signals

In our melanoma study, we identified specific biological signals (cell cycle, keratinization, melanin biosynthesis) and signals from various cells (T cells, B cells, monocytes, endothelial cells, and malignant melanocytes). These signals were strong predictors of patient survival (Dirkse *et al.* 2019). In the glioblastoma study, our method was able to recover the phenotype of tumour cells (proliferative or invasive), highlighted the differences in stable and patient-derived cell lines and supported the main message of the article regarding the benefits of xenograft models over 2D and 3D cell cultures (Golebiewska *et al.* 2020, Supplementary Fig. S2C and D). When working with single-cell RNAseq data, *consICA* allows extracting two components linked to the cell cycle, thus naturally identifying cycling cells and cleaning other signals from this effect (Dirkse *et al.* 2019).

### 3.3 *consICA* for multi-omics and multi-modal data integration

We showed that ICA deconvolution could be used for the integration of mRNA and microRNA data. We detected modules of independent components from both modalities that are united in correlated clusters (Dirkse *et al.* 2019). This could potentially be used for annotating components: projecting functional annotation from the gene level onto miRNA or methylation CpG sites (Scherer *et al.* 2020, Nazarov and Kreis 2021). Recently, we also applied *consICA* to the entire pan-cancer TCGA dataset: mRNA, microRNA, and DNA methylation datasets of 33 cancers were decomposed by *consICA* into 100 independent components each. Correlated components were connected, resulting in the graph in Supplementary Fig. S3C. We identified components linked to increased risk in survival (e.g. cell cycle, keratinization/cornification, inflammation, increased glycolysis, cell motility, and angiogenesis) and checked which modality gives better survival predictors (Supplementary Fig. S3A and B). The weights of purified signals extracted by *consICA* deconvolution could further be used to integrate the omics data and histopathological images, similar to Ash *et al.* (2021).

## 4 Conclusion

The developed R/Bioconductor package *consICA* is an efficient and robust tool for the identification of biologically meaningful omics signals and their interpretation. In the previous publications, we demonstrated the application of *consICA* algorithms to large datasets at various levels: from single-cell to bulk-sample omics, reducing data dimensionality without losing biologically interpretable information. The tool successfully disentangles technical effects from biological signals. Additionally, it allows the integration of data obtained for several modalities: mRNA, miRNA, methylation, and histopathological images.

## Supplementary Material

vbae102_Supplementary_Data

## Data Availability

All datasets referenced in the Results section are publicly available. The data of SEQC project and pan-cancer TCGA dataset were obtained using *seqc* and *curatedTCGAData* R/Bioconductor packages respectively. Glioblastoma bulk-sample microarray data (Golebiewska *et al.* 2020) and single-cell RNA-seq data (Dirkse *et al.* 2019) are available from GEO database under accession numbers GSE134470 and GSE128195. Melanoma RNA-seq data (Nazarov *et al.* 2019) are available under accession number GSE116111.
