# Supplementary material for: consICA: an R package for robust reference-free deconvolution of multi-omics data
Source: Bioinform Adv. 2024 Jul 13;4(1):vbae102. doi: 10.1093/bioadv/vbae102 (PMC11257712; doi:10.1093/bioadv/vbae102)
Supplement: vbae102_Supplementary_Data [file vbae102_supplementary_data.pdf]

Supplementary Data

Content

Supplementary Figure S1.. 2

Supplementary Figure S2.. 3

Supplementary Figure S3.. 4

Supplementary Table 1... 6

Supplementary Text 1.. 8

Supplementary Figure S1

A

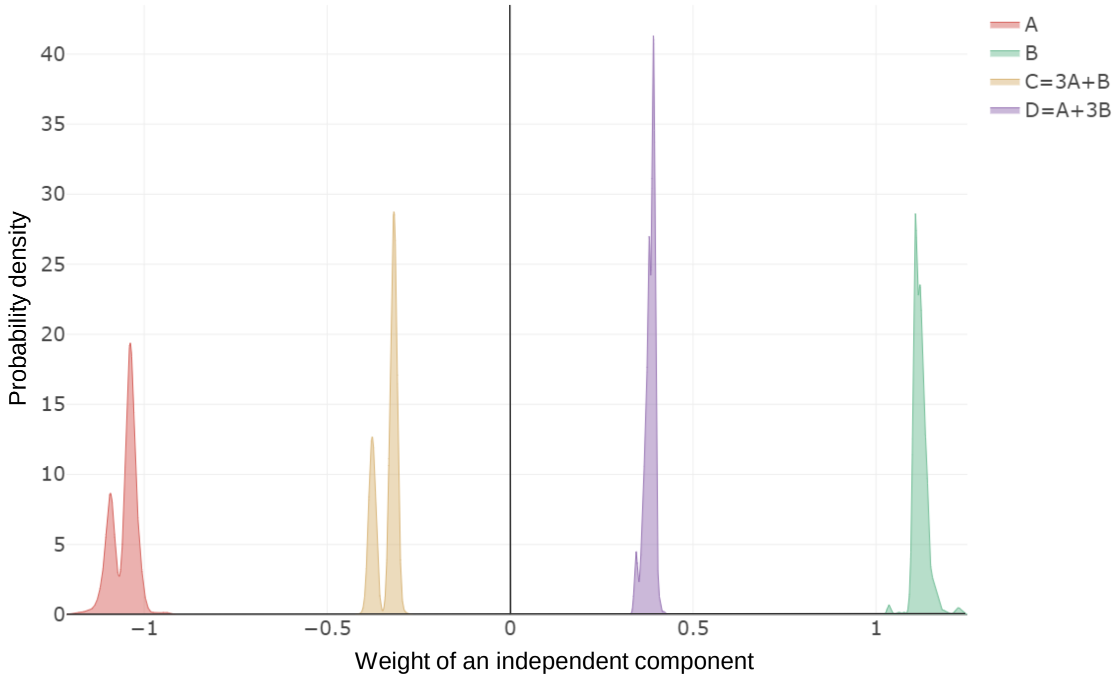

B

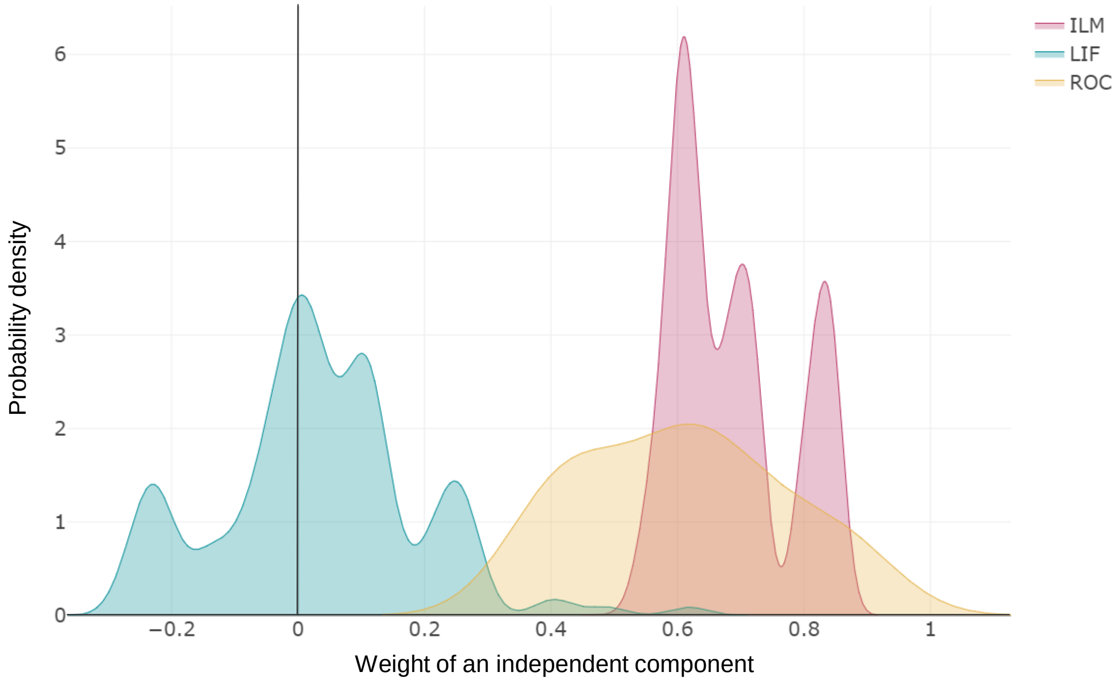

Distribution of weights after ICA-based 3-component deconvolution of SEQC data (2352 samples). In SEQC, RNA samples A and B were mixed in proportions 3:1 and 1:3 forming samples C and D respectively.
(A) Shows the weight coefficient of the component linked to sample composition.
(B) Another component captured differences between experimental platforms (ILM – Illumina HiSeq, LIF – Life Technologies SOLiD, ROC – Roche 454)

Supplementary Figure S2

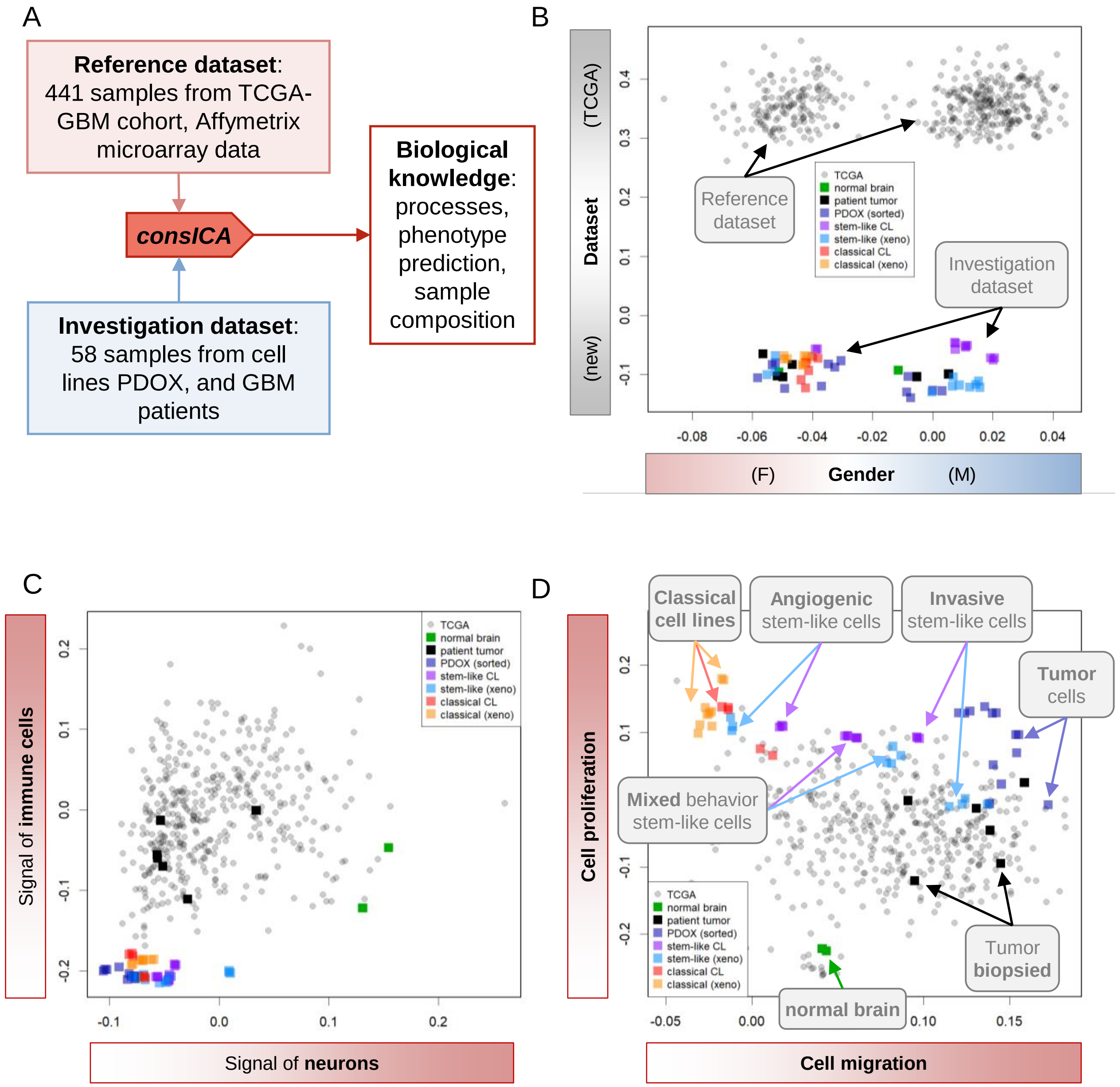

Method validation in a cell-line experiment.
(A) General experiment setting: TCGA-GBM is used as a reference dataset; in-house data from GBM cell lines and patients is an investigation dataset (adapted from https://doi.org/10.1007/s00401-020-02226-7). The following figures (B-C) are given in the coordinates representing the weights of the selected independent components.
(B) Two components captured technical batch effect between datasets and patient's gender.
(C) Biological signals originating from immune cells and neurons help highlight sample compositions: immune cells and neurons are present only in patient biopsies (cancer cases or normal brain) but not in cultivated cells or patient-derived orthotopic xenografts (PDOX). Mouse cells of stroma were sorted out in PDOX experiments.
(D) Prediction of the phenotypic states of the cells. Classical immortal cell lines (CL) U87 and U251 show the highest proliferation but lost their ability to migrate and populate parenchyma. Angiogenic, mixed and invasive stem-like cell lines were correctly allocated on the graph. The highest motility was observed in patient biopsies and preserved in invasive stem-like cells and tumor cells in PDOX models.

Supplementary Figure S3

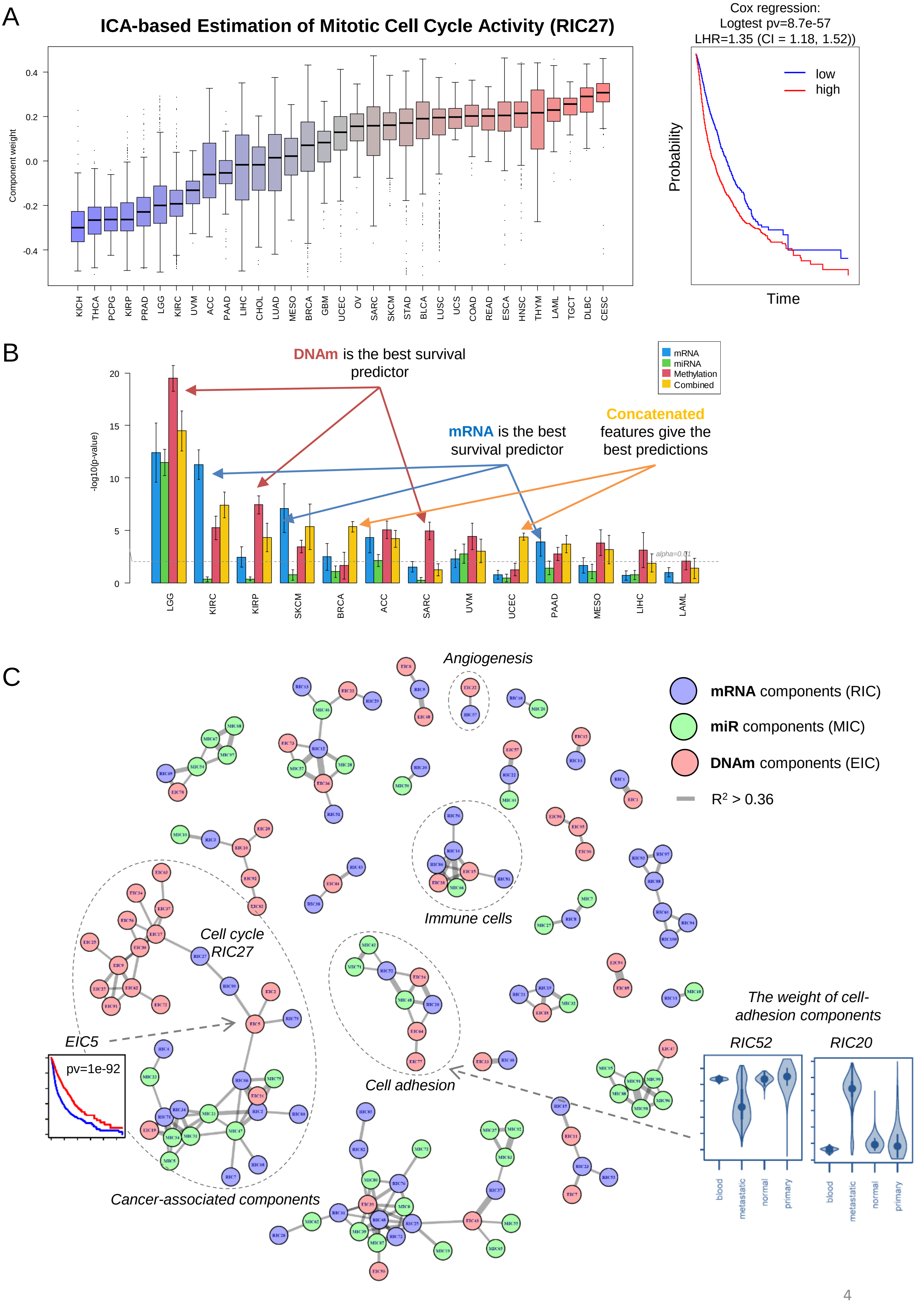

See the caption on the next page

Application of consICA to TCGA – a pan-cancer multi-omics dataset. Three omics levels (mRNA, DNA methylation, and microRNA) were deconvolved into 100 components, and integrated. Components are annotated as follows: RIC1-100 – transcriptomic, mRNA components, EIC1-100 – epigenetic, DNA methylation (DNAm) components, MIC1-100 – microRNA (miR) components.
(A) The component linked to the cell cycle shows average proliferation activity in different tumor biopsies. This component RIC27 is also linked to patient survival (general consideration, not corrected for cancer effect).
(B) Significance of the survival prediction for different cancers using ICA weights of 3 omics levels as features (i.e. corrected for cancer effect). A simple concatenation of the features was tested as well.
(C) Integrated multi-omics network of the components. Several hallmarks of cancer were identified. The large cluster of the components covering all three omics levels originated from the changes in the cancer cells. Among others, it included the cell cycle RIC27 component and epigenetic EIC5 component – the best survival predictor in the TCGA dataset.

Supplementary Table 1

Method	Language	Summary	Advantages	Limitations	Is ICA stable	Annotation/downstream analysis provided	Integration	Ref
fastICA	MAT-LAB, Python, R, others	Basic ICA implementation	Fast and efficient. Suitable for large datasets.	Sensitive to initial conditions, may converge to local optima	No	No	Integrates with various data analysis frameworks and pipelines as a usual R/Python/MATLAB package	1.1
MineICA	R	Framework for the storage and study of ICA decompositions in transcriptomic data	Build-in annotation of independent components	Gene expression data only	No	Gene enrichment analysis, correspondence between independent components with correlation-based graphs, association with sample variables, visualization	Could be integrated into pipelines	1.2
consICA	R	Package for consensus ICA, efficiently extracts biologically meaningful signals from omics data, enabling patient stratification and multimodal integration	Multi-omics, application for different tasks confirmed in publications, build-in annotation, and visualization. Published in Bioconductor.	Functional annotation is applicable only to gene expression data	Yes	Enrichment analysis, survival analysis, Association with sample variables, visualization, automatic reporting	Could be easily integrated into pipelines, compatible with the Bioconductor - works with matrices, SummarizedExperiment and Seurat objects.	1.3
Robustica	Python	Robust ICA with clustering approach for identifying robust components	Multi-omics, extensive customization options	Lack of validation examples on different omics data; Customization of parameters is not be clear	Yes	Number of components estimation	Could be integrated in pipelines, build on scikit-learn library	1.4
BIODICA	Python	Integrated computational environment for ICA application and interpretation with Python *stabilized-ica* package [1.7] in core	Multi-omics, build-in annotation and visualization, GUI, interactive HTML-based reports	Requires Java; Currently mainly applied to transcriptomics data	Yes	Enrichment analysis, Association with sample variables, OFTEN analysis, NaviCell, RBH graphs	stabilized-ica method could be used in Python pipelines	1.5
deconICA	R	Deconvolution of transcriptome through Immune Component Analysis	Build-in annotation and visualization for cell types investigation	Focused on immune cell types deconvolution for gene expression data only; Requires MATLAB or Docker for stabilized algorithm	Available	Number of components estimation, liking components with cell types, correlation of components with LM22 cell profiles [1.8] or Biton et al. [1.9] metagenes, visualisation	Could be used in Python pipelines	1.6

References (Supplementary Table 1)

1.1 Hyvarinen, A. and Oja, E. (2000) Independent component analysis: algorithms and applications. *Neural Networks,* 13(4-5):411-430.

1.2 Biton, A. (2024). MineICA: Analysis of an ICA decomposition obtained on genomics data. R package version 1.44.0. https://www.bioconductor.org/packages/release/bioc/html/MineICA.html.

1.3 Nazarov, P., Kaoma, T., Chepeleva, M. (2024). consICA: consensus Independent Component Analysis. *R package version 2.2.0*. https://www.bioconductor.org/packages/release/bioc/html/consICA.html.

1.4 Anglada-Girotto, M. et al. (2022) robustica: customizable robust independent component analysis. *BMC Bioinformatics,* **23**, 519.

1.5 Captier N. et. al. (2022) BIODICA: a computational environment for Independent Component Analysis of omics data, *Bioinformatics*, **38**, 10, 2963–2964.

1.6 Czerwinska, U. (2018). UrszulaCzerwinska/DeconICA: DeconICA first release (v0.1.0). Zenodo.

1.7 Captier, N et al. (2022) *stabilized-ica*. Python package version 2.0.0. https://github.com/ncaptier/stabilized-ica.

1.8 Newman, A.M. et al. (2015) Robust enumeration of cell subsets from tissue expression profiles*. Nat Methods*, **12**, 5, 453-457.

1.9 Biton, A., et al. (2014) Independent component analysis uncovers the landscape of the bladder tumor transcriptome and reveals insights into luminal and basal subtypes. *Cell Rep.* **20**, 9, 4, 1235-45.

Supplementary Text 1

Here, we aimed to compare the running times of various Independent Component Analysis deconvolution tools for omics data analysis. However, a direct comparison is challenging due to the specific features and intended uses of each method. Our goal was to estimate the running time for general understanding without delving into the biological validity of the extracted independent components.

The testing dataset consisted of SKCM TCGA RNA-seq data comprising 472 samples and 16579 filtered genes, as we used in Nazarov et al., 2019. The decomposition was done into 20 and 40 components.

Since all methods include the *fastICA* algorithm, we used the default parameters from the original package for consistency. For stabilized methods, we set the number of runs to 40. For methods that perform ICA only once, we simulated a loop of 40 repetitions. Stabilized tools inherently spend additional time and resources to combine the results of multiple runs into the final robust decomposition. For stable methods we used 4 cores.

The testing was conducted on a computer with the following specifications:

Processor: Intel(R) Core(TM) i7-10610U CPU @ 2.30 GHz,
RAM: 16.0 GB
Operating system: Windows 10
R Version: 4.3.2
Python Version: 3.11.5

See the results in Table on next page.

Table – running time comparison

Tool	Version	Language	Average Running Time (seconds)		Is ICA stable	Testing details	Explanation of Method Performance	Ref
			20 components	40 components				
fastICA (R package)	1.2-4	R	227	472	No	Run 40 times in a loop; no additional calculations for results combining.	Fast due to minimal data processing; no parallelization	2.1
consICA	2.1.0	R	151	504	Yes	Run on 4 cores; stabilized, performs additional calculations for robust results combing	Perform stabilization steps; accelerate *fastICA* algorithm with *Rfast* [2.7] matrix multiplication; provides reliable and stable results	2.2
MineICA	1.42.0	R	334	604	No	Run 40 times in a loop; no additional calculations for results combining	Runs *fastICA* with additional data transformation. no parallelization	2.3
fastICA (*scikit-learn* package)	1.3.0	Python	38	48	No	Run 40 times in a loop; no additional calculations for results combining	Fast due to minimal data processing; no parallelization	2.4
stabilized-ica	2.0.0	Python	9.8	34	Yes	Run on 4 cores; stabilized and fast	Efficiently combines speed and robustness; optimized for stable outputs	2.5
robustica	0.1.3	Python	> 2880 *	> 3540*	Yes	Run partially (ICA was performed) with whitening step off	Generally robust but not optimized	2.6

(*) When running *robustica* as per provided example, we encountered an error. To circumvent this without altering the package code, we disabled the whitening step, which should reduce the running time. Despite this Otherwise after the step of multiple ICA running on 'dimension reduction' step we got an error. Unfortunately we were not able to run the algorithm completely, but partial run, we can infer that the actual running time would be longer than our estimated time until the error.

We observed a significant difference in the performance of *fastICA* algorithms between R and Python, which explains the shorter running time of *stabilized-ica.* Among the R packages, *consICA* provides a good balance of speed and robustness, making it suitable for reliable omics data analysis. For a smaller number of components, calculation optimization in *consICA* yields better performance compared to fastICA despite the additional calculations required to combine multiple results into one decomposition. In contrast, *MineICA* and *robustica* performed slower, making them less attractive to users seeking stable ICA solutions.

References (Supplementary Text 1)

2.1 Marchini, J.L. et al. (2023). fastICA: FastICA Algorithms to Perform ICA and Projection Pursuit. R package version 1.2-4. https://cran.r-project.org/web/packages/fastICA/index.html.

2.2 Nazarov, P., Kaoma, T., Chepeleva, M. (2024). consICA: consensus Independent Component Analysis. R package version 2.2.0. https://www.bioconductor.org/packages/release/bioc/html/consICA.html.

2.3 Biton, A. (2024). MineICA: Analysis of an ICA decomposition obtained on genomics data. R package version 1.44.0. https://www.bioconductor.org/packages/release/bioc/html/MineICA.html.

2.4 Pedregosa et al. (2011) Scikit-learn: Machine Learning in Python. *JMLR* 12, 2825-2830.

2.5 Captier, N. et al. (2022) *stabilized-ica*. Python package version 2.0.0. https://github.com/ncaptier/stabilized-ica.

2.6 Anglada-Girotto, M. et al. (2022) robustica: customizable robust independent component analysis. *BMC Bioinformatics,* **23**, 519.

2.7 Papadakis,M. et al. (2023). Rfast: A Collection of Efficient and Extremely Fast R Functions. R package version 2.1.0. https://cran.r-project.org/web/packages/Rfast/index.html.
